# Early versus deferred intravenous calcium in patients with sepsis and ionized hypocalcemia: a target-trial emulation

**DOI:** 10.1186/s40560-026-00953-9

**Published:** 2026-10-13

**Authors:** Yuhang Zhang, Guoying Liao, Chenghao Liu, Guanyu Chen, Jianli Song, Yinsheng Liao, Lulu Zhang

**Affiliations:** 1 Department of Anesthesiology, The Second Affiliated Hospital of Chongqing Medical University, Chongqing, 400010 China; 2 Department of Anesthesiology, Zigong Fourth People’s Hospital, Zigong, 643000 Sichuan China; 3 Computer Center, Zigong Fourth People’s Hospital, Zigong, 643000 Sichuan China

**Keywords:** Sepsis, Ionized hypocalcemia, Intravenous calcium, Vasopressors, Target-trial emulation

## Abstract

**Background:**

Intravenous calcium is frequently administered during sepsis, yet whether early treatment improves 24-h hemodynamic recovery remains uncertain. We compared 24-h hemodynamic recovery between calcium initiation within 2 h of a non-emergency low-ionized calcium (iCa) result and no calcium initiation during the first 6 h after the result.

**Methods:**

We conducted a retrospective target-trial emulation of 2016–2025 high-resolution electronic health records from Zigong Fourth People's Hospital. Adults with sepsis, final or corrected iCa of 0.90–1.10 mmol/L, and no emergency indication were eligible at result availability. Strategies were executed calcium within 2 h versus no calcium through 6 h; care after 6 h was unrestricted. Clone–censor weighting and overlap tilting addressed grace periods, measured time-varying confounding, and clinical equipoise. The primary outcome was being alive and vasopressor-free at 24 h. Estimates and two-sided 95% percentile intervals came from 2,000 patient-level bootstrap samples with model refitting.

**Results:**

Of 5,993 patients, 1,538 initiated calcium within 2 h, 147 initiated at 2–6 h, and 4,308 received none through 6 h; none of the latter initiated calcium during 6–24 h. The standardized probability of 24-h hemodynamic recovery was 69.9% with early calcium and 61.1% with deferral, corresponding to a risk difference of + 8.69 percentage points (95% percentile interval, + 4.66 to + 12.84) and a risk ratio of 1.142 (1.074–1.214). The estimated association varied with baseline norepinephrine-equivalent dose (Holm-adjusted *P* = 2.00 × 10⁻⁶), whereas the iCa interaction did not remain statistically supported after adjustment (*P* = 0.097). Early calcium was associated with a 1.29-percentage-point higher observed risk of hypercalcemia (95% percentile interval, + 0.64 to + 2.05). Intervals for severe arrhythmia, new renal replacement therapy, and hospital mortality included the null.

**Conclusions:**

Early intravenous calcium was associated with a higher probability of 24-h hemodynamic recovery than the deferred strategy, whose observed pathway involved no calcium through 24 h. Estimated effects were larger at higher vasopressor doses. Early calcium was associated with a modestly higher observed risk of hypercalcemia. These findings support randomized evaluation in vasopressor-treated patients. Baseline iCa modification remains exploratory.

**Supplementary Information:**

The online version contains supplementary material available at https://doi.org/10.1186/s40560-026-00953-9.

## Background

Sepsis is life-threatening organ dysfunction caused by a dysregulated host response to infection and remains a major cause of death and resource use worldwide [1, 2]. Early treatment focuses on infection control and restoration of tissue perfusion, yet circulatory failure often persists despite fluids and vasoactive drugs [3]. Potentially reversible metabolic abnormalities are, therefore, frequently corrected during resuscitation, although the balance between physiological plausibility and patient-centered benefit is not always established.

Ionized calcium (iCa) is the biologically active calcium fraction and contributes to myocardial contraction, vascular tone, neuromuscular function, and coagulation. Hypocalcemia is reported in approximately 55–85% of intensive care unit patients and is particularly common in sepsis [4, 5]. Its causes are multifactorial, including inflammation-mediated alterations in parathyroid hormone signaling, vitamin D metabolism, renal function, acid–base balance, and citrate exposure [4–6]. Lower iCa tracks with illness severity, but large cohorts suggest that prognostic risk is concentrated at more extreme values and may be nonlinear [7, 8]. Because this association is strongest at extreme values rather than uniform across the hypocalcemic range, mild-to-moderate hypocalcemia may partly reflect the severity of the host response rather than a uniformly modifiable cause of organ dysfunction. Prognostic associations alone, therefore, cannot establish that correcting these values improves clinical outcomes.

The physiological role of calcium makes early intravenous administration attractive in vasopressor-treated patients. However, extracellular hypocalcemia may coexist with intracellular calcium overload during sepsis; additional calcium could amplify inflammation, endothelial injury, and cellular dysfunction [4, 9]. Clinical evidence remains sparse and conflicting. A MIMIC-III analysis suggested that associations between calcium supplementation and mortality varied with baseline iCa and organ-failure severity, but treatment was defined over the admission and analyzed using conventional propensity-score methods [10]. A recent single-center cohort found no improvement in shock resolution or survival and possible harm among patients with sepsis and mild hypocalcemia [11]. No randomized trial has evaluated calcium replacement for hypocalcemia in critically ill adults [4, 5]. Evidence from another resuscitation syndrome also cautions against treating hemodynamic rationale as proof of clinical benefit: a randomized trial in out-of-hospital cardiac arrest found no benefit from calcium [12]. This uncertainty concerns non-emergency supplementation; calcium remains appropriate for recognized indications, such as severe symptomatic hypocalcemia and major transfusion-associated hypocalcemia [5, 13].

The central unresolved question is, therefore, not whether low iCa predicts poor outcome, but when intravenous calcium should be given after a non-emergency low-iCa result. Previous studies have not aligned eligibility, treatment assignment, and follow-up at the qualifying measurement, leaving them vulnerable to confounding by evolving shock severity and time-related bias. Target-trial emulation provides a framework for defining time zero and comparing treatment-timing strategies in longitudinal clinical data [14, 15]. We, therefore, emulated a target trial to compare 24-h hemodynamic recovery under intravenous calcium initiated within 2 h versus no calcium through 6 h among adults with sepsis and iCa 0.90–1.10 mmol/L; care after 6 h was unrestricted. We also examined whether estimated strategy differences varied with baseline iCa and hemodynamic severity.

## Methods

### Study design and data source

**Fig. 1:**
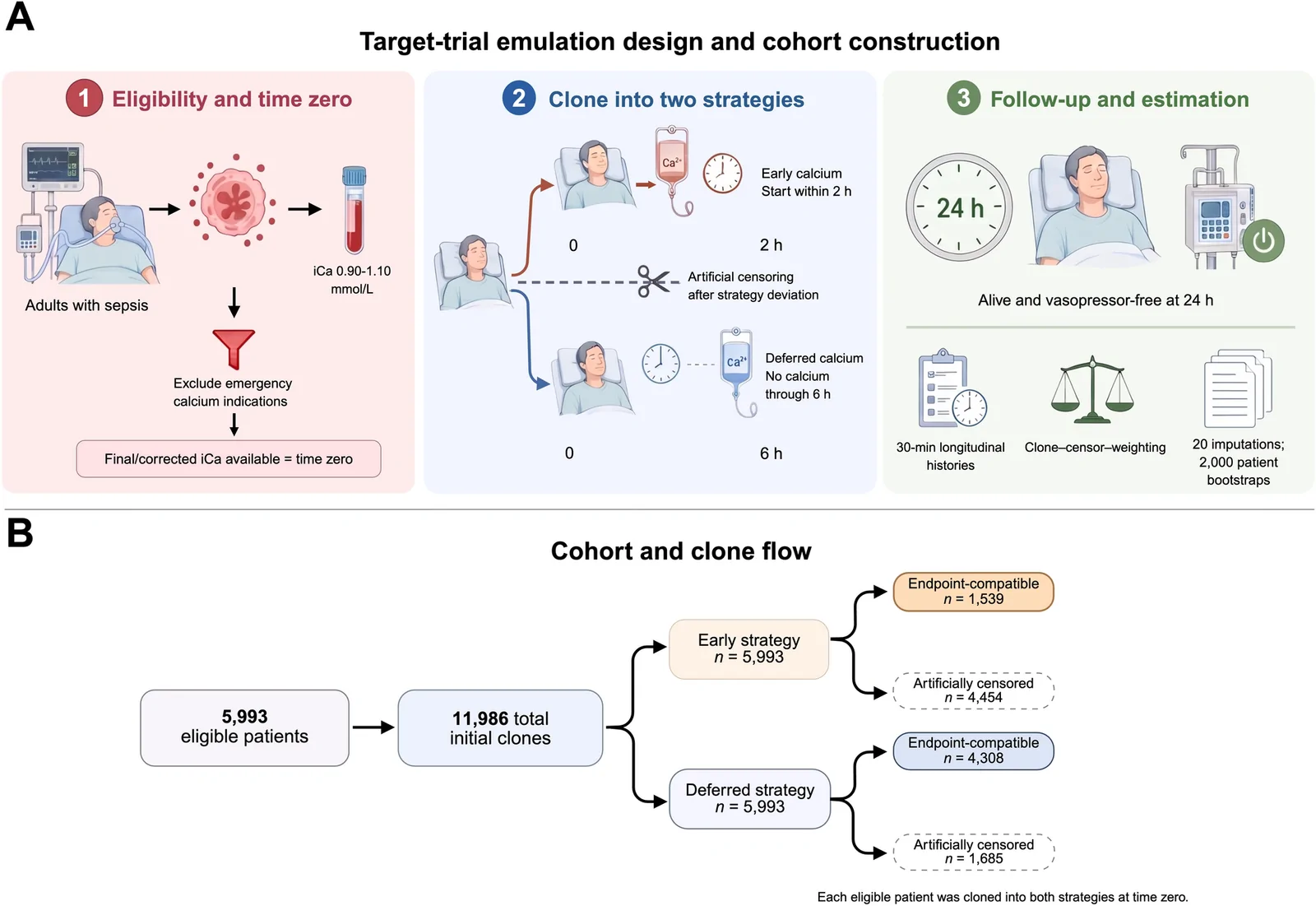
Target-trial emulation design and cohort construction. **A** Adults with sepsis became eligible when a final or corrected ionized calcium result of 0.90–1.10 mmol/L became available, after exclusion of emergency calcium indications. At time zero, each patient was cloned into an early calcium strategy (intravenous calcium initiated within 2 h) and a deferred calcium strategy (no intravenous calcium through 6 h; care after 6 h unrestricted); clones were artificially censored at strategy deviation. Follow-up ended at 24 h, when hemodynamic recovery was defined as being alive and free of positive-rate vasopressor infusion. Treatment histories were updated every 30 min. Primary inference used 2,000 patient-level bootstrap replicates with model refitting; 20 chained-equation imputations provided complementary inference. **B** Flow of 5,993 eligible patients and 11,986 initial clones to endpoint compatibility or artificial censoring. None of the 4,308 deferred-pathway patients initiated calcium during 6–24 h. Ca^2^⁺, calcium ion; iCa, ionized calcium. Figure 1 was created entirely in BioRender (https://BioRender.com/t0938zh)

We conducted a retrospective target-trial emulation using routinely collected, high-resolution electronic health-record data from adult intensive care units (ICUs) at Zigong Fourth People's Hospital, a tertiary teaching hospital in China, between 2016 and 2025. The Ethics Committee of Zigong Fourth People's Hospital approved the study (EC2026082) and waived informed consent because the study used retrospectively collected clinical data. The hypothetical trial and its observational emulation were specified before formal outcome modeling, with eligibility, treatment assignment, and follow-up aligned at a common time zero [14–16] (Fig. 1). The protocol was not registered in a public registry. The complete target-trial specification is provided in Table S1 (Additional file 1), and data sources, look-back windows, and operational definitions are detailed in Table S2 (Additional file 1).

### Eligibility and time zero

Eligible patients were aged 18 years or older and had operational sepsis, defined as evidence from at least two of four infection categories during the preceding 72 h (executed systemic antibiotics, microbiological sampling, structured suspected infection, or an infection diagnosis) together with a Sequential Organ Failure Assessment (SOFA) score of at least 2, calculated from information available during the preceding 24 h [1, 17]. The index event was a final or corrected ionized calcium (iCa) result of 0.90–1.10 mmol/L during an ICU stay. Time zero was the result-availability time, not specimen collection.

We excluded events preceded by executed intravenous calcium within 6 h or by a strong emergency indication: potassium at least 6.0 mmol/L within 2 h; cardiac arrest, cardiopulmonary resuscitation, cardiopulmonary bypass, or a massive-transfusion protocol within 6 h; calcium-channel-blocker poisoning within 24 h; or active citrate-anticoagulated renal replacement therapy (RRT), extracorporeal membrane oxygenation, or other mechanical circulatory support. A treatment-support audit based on exposure timing and covariates was completed before formal outcome modeling and did not use outcomes. Because repeat eligibility did not support both strategies adequately, the formal estimand used each patient’s first fully eligible event; repeated entries were retained only as an identifiability diagnostic.

### Treatment strategies and cloning

The early strategy was initiation of executed, non-RRT intravenous calcium gluconate or calcium chloride from time zero through 2 h. The deferred strategy was not executed non-RRT intravenous calcium before 6 h; initiation at exactly 6 h remained compatible. Subsequent calcium after 6 h was neither required nor prohibited. The 2-h window represented an immediately actionable response to a reported laboratory result. The 6-h boundary was an operational definition of deferral, separating immediate treatment from no calcium initiation across repeated 30-min decision intervals; it was not intended as a validated biological or clinical threshold. Exposure-timing distributions were examined without outcomes during the treatment-support audit; they showed an early treatment cluster and relatively few starts during 2–6 h. The windows were then fixed before outcome modeling. Medication orders without documented administration did not define treatment.

At time zero, each patient was cloned into both strategies. An early-strategy clone remained compatible during the 2-h grace period and was artificially censored if the patient was alive, but untreated at 2 h. A deferred-strategy clone was censored at the first calcium initiation before 6 h. Treatment between 2 and 6 h, therefore, caused both clones eventually to deviate, but was not excluded before cloning. Death preceding artificial censoring was retained as an outcome failure.

### Outcomes

Follow-up began at time zero. The primary outcome was hemodynamic recovery, defined as being alive and free of any positive-rate norepinephrine, epinephrine, vasopressin, or dopamine infusion exactly 24 h later; death before 24 h was failure. Secondary outcomes were any post-index iCa greater than 1.30 mmol/L within 24 h, a new severe or treatment-requiring arrhythmia within 24 h, new RRT within 24 h among patients not receiving RRT at baseline, and hospital mortality.

### Covariates

All covariates were restricted to information available by the relevant decision time. Baseline variables comprised age, sex, calendar year, a deidentified within-institution administrative location code, ICU type, total SOFA score, index iCa, time since ICU admission, infection-evidence count, same-specimen pH available by time zero, lactate, potassium, creatinine, platelets, bilirubin, albumin, hemoglobin, white-cell count, mean arterial pressure, heart rate, Glasgow Coma Scale score, invasive ventilation, active RRT, hydrocortisone, transfusion, and norepinephrine-equivalent dose (NEE). NEE was calculated using a prespecified formula informed by published equivalence ranges: norepinephrine + epinephrine + 2.5 × vasopressin + dopamine/150 [18]. Current or recent iCa, pH, lactate, potassium, mean arterial pressure, heart rate, NEE, ventilation, RRT, and transfusion status were updated at each interval start. An expanded sensitivity model additionally included magnesium, phosphate, active malignancy, infection-source indicators, left ventricular ejection fraction (LVEF), procalcitonin (PCT), C-reactive protein (CRP), aspartate aminotransferase, and international normalized ratio, with missingness indicators where applicable. Reliable structured data were available for recorded active malignancy, defined by an active diagnosis documented by time zero during the current hospital encounter; this variable was included in the expanded model. Reliable structured data on parathyroid disease, vitamin D status, and bisphosphonate exposure were unavailable.

### Statistical analysis

Time from 0 to 6 h was divided into 30-min intervals. Among patients alive and untreated at each interval start, a pooled logistic model estimated the conditional calcium-initiation hazard from interval time, baseline covariates, selected time interactions, and the time-updated covariates. Inverse observed-path probabilities accounted for artificial censoring. A separate baseline model estimated the probability of early rather than deferred adherence among histories compatible with either strategy. Path weights were multiplied by the overlap tilting function *e(X)*{1 − *e(X)*}, targeting patients with clinical equipoise rather than the whole eligible population [19]. Primary weights were untruncated.

Strategy-specific weighted risks were standardized to the clinical-overlap population. The primary estimand was the 24-h risk difference, in percentage points, for the early calcium strategy versus the deferred calcium strategy. The risk ratio was secondary. A weighted linear probability model estimated the risk difference. Covariate balance was assessed using absolute standardized mean differences, with less than 0.10 prespecified as acceptable. Effective sample size, weights, and predicted-hazard distributions were also examined.

For primary reporting, point estimates were the medians of 2,000 patient-level bootstrap samples, with two-sided 95% percentile intervals. Each sample refitted the treatment-path and overlap models and used deterministic median/mode imputation with missingness indicators. A complementary analysis used 20 chained-equation datasets with predictive mean matching for incomplete continuous baseline covariates [20]. Exposure, time zero, death, and outcomes were not imputed. Observed time-updated measurements replaced carried-forward imputed baseline values. The multiple-imputation analysis used patient-clustered robust variances and Rubin’s rules.

Prespecified sensitivity analyses applied within-strategy weight truncation at the 0.5th/99.5th, 1st/99th, and 2.5th/97.5th percentiles, excluded ICU discharge before the relevant grace-period endpoint, and used 60-min intervals. Effect modification was examined for continuous baseline iCa, same-specimen pH, lactate, and NEE; iCa was modeled with a three-degree-of-freedom natural spline. The four interaction tests were treated as one family and Holm-adjusted. Any iCa zero crossing was considered exploratory. All eligible records were analyzed without an a priori sample-size calculation. Analyses used R version 4.5.2, with data. Table 1.18.2.1, mice 3.19.0, and sandwich 3.1.1. Causal interpretation required consistency, conditional exchangeability given the measured covariate histories, positivity within the clinical-overlap population, and correct specification of the treatment-path and outcome models. Reporting followed the Strengthening the Reporting of Observational Studies in Epidemiology (STROBE) statement, its Reporting of Studies Conducted Using Observational Routinely Collected Health Data (RECORD) extension, and the Transparent Reporting of Observational Studies Emulating a Target Trial (TARGET) statement [21–23].

Baseline iCa and NEE were selected as effect modifiers because they directly represent the biochemical and hemodynamic dimensions of the treatment decision. Same-specimen pH was selected because acid–base status alters calcium binding and iCa, whereas lactate reflects tissue hypoperfusion and shock severity [3–5]. Additional exploratory analyses examined continuous SOFA score, four infection-source indicators, baseline PCT, and pre-index pathogen group. SOFA and PCT were modeled with natural splines. The four infection-source tests formed one Holm family, and the PCT and pathogen tests formed a second family. The expanded-covariate analysis repeated the 2,000-sample patient bootstrap with refitting of both weight models.

## Results

### Cohort and treatment pathways

The source data contained 17,644 eligible decisions separated by at least 6 h. The formal analysis included the first eligible decision from 5,993 unique patients. Within the observed 6-h treatment window, calcium was initiated in 1,538 patients by 2 h and in 147 patients after 2 h, but before 6 h; 4,308 patients had no calcium initiation through 6 h. None of these 4,308 patients initiated calcium during 6–24 h. Thus, although the deferred strategy formally allowed treatment after 6 h, the observed comparator was no calcium through 24 h rather than mandatory later treatment. Cloning produced 11,986 initial clones. At the endpoint, 1,539 early-strategy clones and 4,308 deferred-strategy clones remained compatible, while 4,454 and 1,685 clones, respectively, had been artificially censored (Fig. 1B; Table S3). Among patients receiving calcium within 6 h, the median initiation time was 0.50 h (interquartile range, 0.36–0.68); 1,385 of 1,685 initiations occurred during the first hour (Fig. S1).

Early initiation occurred in 25.7% of first eligible decisions, compared with 2.1–3.2% across decision numbers 2–8. In the repeated-eligibility diagnostic, the early-strategy effective sample size was approximately 61 and the maximum weighted absolute standardized mean difference (SMD) was 0.890. The corresponding values for the first-eligibility analysis were 632 and 0.087 (Fig. S2).

### Baseline characteristics and weighting diagnostics

The mean age of the eligible cohort was 63.4 years (SD, 14.9), 2,624 patients (43.8%) were female, the mean Sequential Organ Failure Assessment score was 6.2 (SD, 2.8), and mean index ionized calcium (iCa) was 0.999 mmol/L (SD, 0.051). Before weighting, early-compatible histories had higher mean SOFA scores than deferred-compatible histories (7.2 versus 5.8), higher mean norepinephrine-equivalent dose (NEE; 0.111 versus 0.063 µg/kg/min), and more frequent invasive mechanical ventilation (40.0% versus 30.1%). The corresponding absolute SMDs were 0.523, 0.372, and 0.208 (Table 1).

**Table 1:** Baseline characteristics and covariate balance before and after clone–censor–weighting

Characteristic	Overall eligible cohort (*N* = 5,993)	Early-compatible histories (*n* = 1,539)	Deferred-compatible histories (*n* = 4,308)	Unweighted absolute SMD	Weighted early strategy	Weighted deferred strategy	Weighted absolute SMD
Demographic characteristics							
Age, years	63.4 (14.9)	63.9 (14.8)	63.2 (14.9)	0.047	64.2	64.0	0.013
Female sex	2,624 (43.8%)	641 (41.7%)	1,916 (44.5%)	0.057	40.9%	43.2%	0.046
Calendar period, n (%)							
2016–2018	1,990 (33.2%)	381 (24.8%)	1,567 (36.4%)	0.254	26.6%	26.3%	0.008
2019–2021	1,628 (27.2%)	449 (29.2%)	1,140 (26.5%)	0.061	25.4%	28.2%	0.065
2022–2025	2,375 (39.6%)	709 (46.1%)	1,601 (37.2%)	0.181	48.0%	45.5%	0.051
Administrative location within institution, n (%)							
Location A	4,277 (71.4%)	1,128 (73.3%)	3,046 (70.7%)	0.058	72.6%	72.2%	0.011
Location B	1,716 (28.6%)	411 (26.7%)	1,262 (29.3%)	0.058	27.4%	27.8%	0.011
ICU type, n (%)							
Coronary care ICU	610 (10.2%)	148 (9.6%)	445 (10.3%)	0.024	9.8%	10.3%	0.018
Emergency ICU	781 (13.0%)	214 (13.9%)	550 (12.8%)	0.033	14.2%	13.4%	0.022
Medical ICU	2,604 (43.5%)	697 (45.3%)	1,836 (42.6%)	0.054	45.3%	45.5%	0.004
Mixed ICU	824 (13.7%)	222 (14.4%)	578 (13.4%)	0.029	13.6%	14.1%	0.015
Neurological ICU	88 (1.5%)	0 (0.0%)	88 (2.0%)	0.204	0.0%	0.0%	0.001
Surgical ICU	1,012 (16.9%)	258 (16.8%)	737 (17.1%)	0.009	17.2%	16.7%	0.013
Trauma ICU	74 (1.2%)	0 (0.0%)	74 (1.7%)	0.187	0.0%	0.0%	0.001
Clinical status at time zero							
SOFA score, points	6.2 (2.8)	7.2 (2.9)	5.8 (2.6)	0.523	7.0	7.0	0.013
Index iCa, mmol/L	0.999 (0.051)	0.996 (0.049)	1.000 (0.052)	0.072	0.998	0.998	0.004
Same-specimen pH	7.336 (0.062)	7.329 (0.063)	7.339 (0.062)	0.163	7.331	7.332	0.017
Lactate, mmol/L	2.6 (1.2)	2.7 (1.2)	2.6 (1.2)	0.055	2.6	2.6	0.014
Creatinine, mg/dL	1.30 (0.63)	1.23 (0.56)	1.32 (0.65)	0.155	1.23	1.24	0.014
Potassium, mmol/L	4.27 (0.29)	4.29 (0.37)	4.27 (0.25)	0.081	4.29	4.29	0.024
MAP, mmHg	49.8 (8.4)	50.4 (8.5)	49.6 (8.3)	0.088	50.1	50.2	0.009
NEE, µg/kg/min	0.077 (0.123)	0.111 (0.141)	0.063 (0.113)	0.372	0.107	0.103	0.027
Inflammatory laboratory tests							
White blood cell count maximum, × 10⁹/L	13.1 (3.7)	13.7 (5.1)	12.9 (3.0)	0.186	13.6	13.5	0.009
C-reactive protein, mg/L	106.9 (74.8)	125.6 (92.4)	100.8 (67.4)	0.307	119.9	116.5	0.040
Procalcitonin, ng/mL	1.51 (2.23)	2.07 (3.07)	1.33 (1.83)	0.291	1.88	1.78	0.039
Liver-related laboratory tests							
Total bilirubin maximum, mg/dL	1.04 (0.62)	1.08 (0.70)	1.03 (0.58)	0.083	1.06	1.07	0.028
Albumin minimum, g/L	29.0 (4.1)	28.2 (5.2)	29.3 (3.5)	0.254	28.6	28.5	0.033
Organ support and recent therapies, n (%)							
Invasive mechanical ventilation	1,969 (32.9%)	616 (40.0%)	1,298 (30.1%)	0.208	38.9%	36.9%	0.042
Active RRT at time zero	306 (5.1%)	55 (3.6%)	246 (5.7%)	0.102	3.9%	3.7%	0.011
Hydrocortisone in the preceding 24 h	585 (9.8%)	152 (9.9%)	412 (9.6%)	0.011	9.8%	10.9%	0.035
Transfusion in the preceding 24 h	432 (7.2%)	94 (6.1%)	329 (7.6%)	0.060	5.0%	5.9%	0.040

Values are mean (SD) or n (%); weighted columns show weighted means or percentages. Absolute SMD below 0.10 indicated acceptable balance. The overall column includes 5,993 first-eligible patients. Strategy-compatible columns contain observed histories remaining compatible through each grace period and are not mutually exclusive; one patient who died during both grace periods contributed to both columns (5,847 histories; 5,846 unique patients). Weighted statistics use the untruncated final CCW–ATO weight (inverse treatment-path weighting with baseline overlap tilting). For this diagnostic display, missing continuous covariates were median-imputed and missingness indicators were retained. Primary estimates used 2,000 patient-level bootstrap resamples; multiple imputation was complementary. CRP and PCT were excluded from the primary weight model, but included in the expanded-covariate sensitivity analysis. Table S7 separately reports the most recent finalized or corrected pre-index laboratory results within 24 h, without imputation, as medians (interquartile ranges); Table S6 reports additional clinical characteristics. Locations A and B are deidentified administrative codes within Zigong Fourth People's Hospital. ATO, average treatment effect in the overlap population; CCW, clone–censor–weight; CRP, C-reactive protein; iCa, ionized calcium; ICU, intensive care unit; MAP, mean arterial pressure; NEE, norepinephrine-equivalent dose; PCT, procalcitonin; RRT, renal replacement therapy; SD, standard deviation; SMD, standardized mean difference; SOFA, Sequential Organ Failure Assessment

Missingness exceeded 25% for same-specimen pH, bilirubin, albumin, and Glasgow Coma Scale score; albumin had the greatest missingness at 40.3% (Table S4). Among endpoint-compatible clones, median untruncated weights were 1.292 for the early strategy and 0.099 for the deferred strategy; the 99th percentiles were 14.64 and 0.781, respectively. Effective sample sizes were 632 and 1,936 (Fig. S3). After weighting, every modeled covariate had an absolute SMD below 0.10 (Fig. S4). The maximum was 0.087 for calendar year 2024; the weighted absolute SMDs for SOFA score, NEE, and invasive ventilation were 0.013, 0.027, and 0.042, respectively (Table S5).

Patients were drawn from seven ICU types and were not restricted to sepsis without comorbidity. In the early and deferred observed pathways, respectively, active malignancy was recorded in 10.2% and 16.9%, heart failure in 11.6% and 16.7%, chronic kidney disease in 9.8% and 15.0%, and cirrhosis in 9.8% and 16.4%. Transthoracic echocardiography was available in 69.6% and 63.3%; median LVEF was 54.6% and 56.0%, and LVEF below 40% was recorded in 7.3% and 5.7% (Table S6).

Pulmonary, urinary, intra-abdominal, and skin or soft-tissue sources were recorded in 27.0%, 15.8%, 24.4%, and 15.0% of the early pathway and 30.6%, 15.8%, 28.8%, and 15.5% of the deferred pathway. In the additional pre-index laboratory extract, values were the most recent finalized or corrected results available within 24 h before time zero and were summarized without imputation. Median magnesium and phosphate were similar, but were available in fewer than 25% of patients. Median aspartate aminotransferase was 42.17 versus 34.04 U/L, bilirubin 1.11 versus 0.91 mg/dL, white blood cell count 11.29 versus 10.40 × 10⁹/L, CRP 106.55 versus 83.93 mg/L, and PCT 1.27 versus 0.82 ng/mL. Bacterial-only pathogens were recorded in 33.6% and 31.1%, while 39 patients had recorded SARS-CoV-2 positivity (Tables S6 and S7).

### Primary outcome and sensitivity analyses

**Fig. 2:**
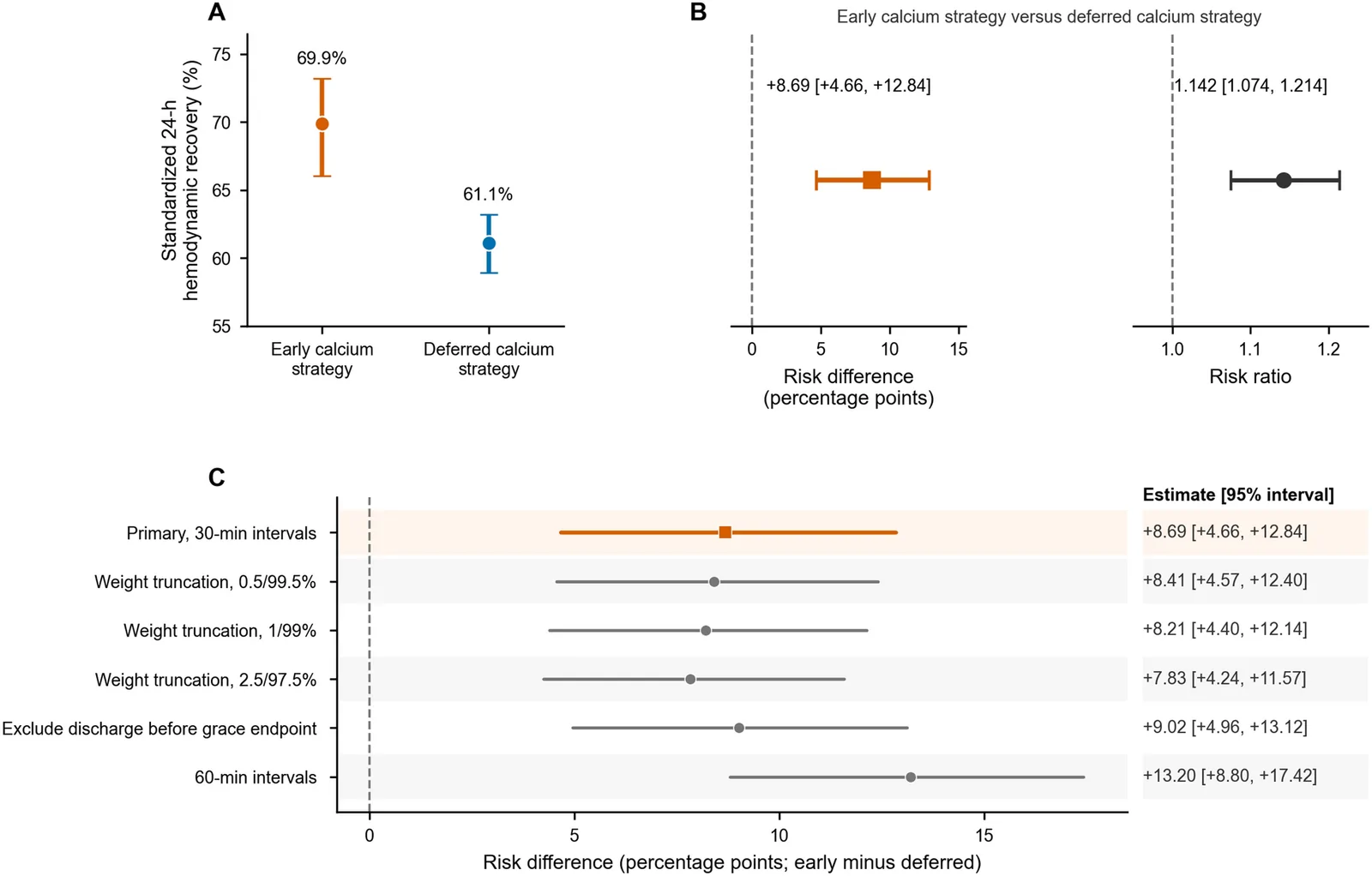
Primary effect and sensitivity analyses. **A** Standardized probabilities of 24-h hemodynamic recovery under the early calcium strategy and deferred calcium strategy. **B** Primary absolute and relative effects comparing early with deferred calcium. **C** Risk differences in the primary analysis and prespecified sensitivity analyses. Points denote patient-bootstrap median estimates and bars denote two-sided 95% percentile intervals from 2,000 patient-level bootstrap replicates with refitting of the treatment-path and baseline-overlap models. Effects pertain to the clinical-overlap population

In the 2,000-replicate patient bootstrap, the standardized probability of 24-h hemodynamic recovery was 69.9% under the early calcium strategy and 61.1% under the deferred strategy. The risk difference was + 8.69 percentage points (95% percentile interval, + 4.66 to + 12.84), and the risk ratio was 1.142 (95% percentile interval, 1.074–1.214) (Fig. 2). All 2,000 bootstrap replicates completed. The median bootstrap effective sample sizes were 588 for the early strategy and 1,875 for the deferred strategy (Fig. S5).

The 20-imputation analysis estimated standardized risks of 69.8% and 60.7%, a risk difference of + 9.11 percentage points (95% CI + 4.94 to + 13.27), and a risk ratio of 1.150 (95% CI 1.080–1.224). The deterministic estimate was + 8.77 percentage points. The expanded-covariate bootstrap estimated risks of 70.1% and 61.2%, a risk difference of + 8.89 percentage points (95% percentile interval, + 5.04 to + 13.03), and a risk ratio of 1.145 (1.082–1.217); the maximum weighted absolute SMD was 0.090 (Table S8). Across prespecified weight-truncation analyses, risk differences ranged from + 7.83 to + 8.41 percentage points. Excluding ICU discharge before the relevant grace-period endpoint yielded + 9.02 percentage points (95% percentile interval, + 4.96 to + 13.12). With 60-min rather than 30-min intervals, the risk difference was + 13.20 percentage points (95% percentile interval, + 8.80 to + 17.42); the maximum weighted absolute SMD was 0.080 and the early-strategy effective sample size was 353 (Fig. S6). All prespecified sensitivity analyses produced risk-difference estimates in the same direction as the primary analysis. The larger 60-min estimate indicated that effect magnitude was sensitive to time discretization.

### Effect modification

**Fig. 3:**
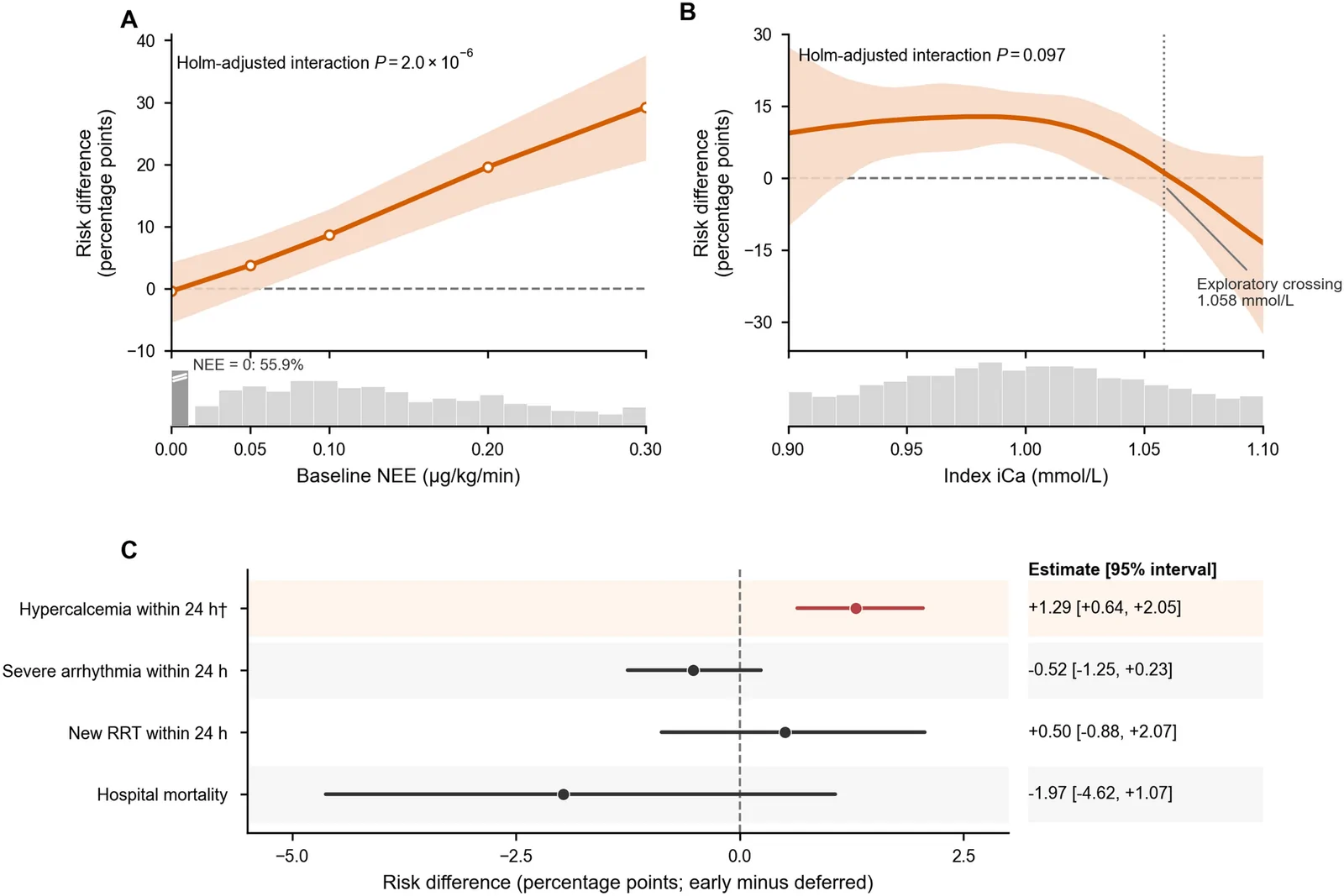
Effect heterogeneity and clinical trade-offs. **A** Adjusted risk difference for 24-h hemodynamic recovery across baseline norepinephrine-equivalent dose. The support distribution displays the exact NEE = 0 mass separately from positive-dose bins; 3,351 of 5,993 patients (55.9%) had NEE = 0. **B** Adjusted risk difference across index ionized calcium; the vertical dotted line marks the conditional median zero crossing among 1,903 of 2,000 bootstrap replicates with an in-range crossing and does not denote a validated treatment threshold. The histogram shows the index-iCa distribution among 5,993 first-eligible patients. **C** Absolute risk differences for secondary and safety outcomes, calculated as early minus deferred. Curves and points denote patient-bootstrap median estimates; bands and intervals denote two-sided 95% percentile intervals from 2,000 patient-level bootstrap replicates. Interaction tests for NEE, iCa, same-specimen pH, and lactate were adjusted as one family using the Holm method; only NEE remained statistically supported after correction. The dagger identifies hypercalcemia, for which post-index iCa was observed in 85.8% of early-strategy and 78.3% of deferred-strategy clones. iCa, ionized calcium; NEE, norepinephrine-equivalent dose; RRT, renal replacement therapy

The association between strategy and hemodynamic recovery varied across baseline NEE (Holm-adjusted *P* = 2.00 × 10⁻⁶) (Fig. 3). Estimated risk differences were − 0.42 percentage points (95% percentile interval, − 5.48 to + 4.24) at NEE 0, + 8.64 (+ 4.29 to + 12.81) at 0.10 µg/kg/min, + 19.58 (+ 13.60 to + 25.26) at 0.20 µg/kg/min, and + 29.24 (+ 20.69 to + 37.57) at 0.30 µg/kg/min. For index iCa, the unadjusted and Holm-adjusted interaction P values were 0.032 and 0.097, respectively; Holm-adjusted P values were 1.000 for same-specimen pH and lactate (Table S9).

The iCa curve had positive point estimates across most of the central eligible range and declined near its upper boundary. Estimated risk differences were + 12.26 percentage points (95% percentile interval, + 4.94 to + 19.12) at iCa 0.95 mmol/L, + 12.39 (+ 6.96 to + 17.81) at 1.00 mmol/L, + 3.81 (− 4.00 to + 10.48) at 1.05 mmol/L, and − 13.48 (− 32.50 to + 4.78) at 1.10 mmol/L. An in-range crossing occurred in 1,903 of 2,000 bootstrap replicates; the conditional median was 1.058 mmol/L (95% percentile interval, 0.903–1.086) (Fig. S7). Because the iCa interaction did not remain supported after Holm adjustment, the iCa-specific estimates and crossing were treated as exploratory.

In exploratory analyses, the strategy association varied across SOFA score (*P* = 3.62 × 10⁻4); estimated risk differences were + 2.61 percentage points at SOFA 6, + 14.30 at SOFA 8, and + 21.87 at SOFA 10. None of the pulmonary, urinary, intra-abdominal, or skin or soft-tissue source interactions remained supported after Holm adjustment (all *P* ≥ 0.219). Interactions with PCT and pre-index pathogen group were also unsupported (Holm-adjusted P = 0.386 for each) (Table S9; Fig. S8).

### Secondary and safety outcomes

Hypercalcemia within 24 h occurred in 22 early-compatible and five deferred-compatible clones. Standardized risks were 1.38% and 0.08%, corresponding to a risk difference of + 1.29 percentage points (95% percentile interval, + 0.64 to + 2.05). Post-index iCa was observed in 85.8% of early-strategy clones and 78.3% of deferred-strategy clones. Risk differences were − 0.52 percentage points (95% percentile interval, − 1.25 to + 0.23) for severe arrhythmia, + 0.50 (− 0.88 to + 2.07) for new renal replacement therapy, and − 1.97 (− 4.62 to + 1.07) for hospital mortality; each interval included zero (Table S10).

## Discussion

This target-trial emulation addresses a practical treatment decision that remains unresolved in critical care: whether a non-emergency iCa result of 0.90–1.10 mmol/L should prompt early intravenous calcium during sepsis. In the clinical-overlap population, calcium initiation within 2 h was associated with an 8.69-percentage-point higher probability of being alive and vasopressor-free at 24 h than the deferred strategy. The formal deferred strategy required no calcium through 6 h, but no deferred-pathway patient initiated calcium during 6–24 h. The observed contrast, therefore, compared early calcium with no calcium through 24 h, not routine later replacement. Estimated differences increased with baseline NEE. The iCa curve declined near the upper eligibility boundary, although the adjusted interaction was not statistically supported. Together, these findings suggest a trial-enrichment framework centered on vasopressor-treated patients with low iCa.

Calcium replacement in the ICU is currently guided by physiological rationale, local protocols, and individual preference. A recent multicenter study of 47,988 critically ill adults documented substantial hospital-level variation in electrolyte supplementation and called for randomized evaluation of treatment thresholds [24]. The evidence gap is longstanding: a systematic review found no controlled ICU trial capable of determining whether parenteral calcium improves mortality, organ function, or other patient outcomes [25]. Earlier cohorts established that low iCa is common and tracked with illness severity, neuroendocrine disruption, and death [26, 27], yet prognostic association alone cannot identify who benefits from replacement. The two recent sepsis cohorts also produced discordant severity-dependent associations [10, 11]. Our study examines a more immediate decision than admission-level or episode-level calcium exposure. Alignment at the qualifying laboratory result, explicit 2-h and 6-h strategies, and a proximal circulatory endpoint focus the analysis on the decision point at which supplementation might alter vasopressor dependence.

The hemodynamic pattern is biologically coherent. Ionized calcium is directly related to arterial pressure in critically ill patients, and hypocalcemia is more frequent among those requiring vasopressors [28]. Small physiological studies have shown rapid increases in contractility, arterial pressure, or systemic vascular resistance after intravenous calcium, although responses varied with the underlying cardiovascular state and calcium exposure [29, 30]. During septic vasoplegia, receptor desensitization, excess nitric oxide signaling, and disruption of calcium-dependent vascular smooth-muscle contraction reduce responsiveness to catecholamines [31, 32]. Sepsis-induced myocardial dysfunction may further narrow cardiovascular reserve [33]. Against this background, the NEE interaction is the principal effect-modification finding. The near-null estimate without vasopressors and the progressively larger differences at NEE 0.10, 0.20, and 0.30 µg/kg/min are compatible with effect modification by hemodynamic severity. A state-dependent biological response is one plausible interpretation. Residual confounding by shock trajectory, treatment preference, and incompletely recorded bedside assessment is another, particularly at higher vasopressor doses.

The iCa curve provides an exploratory second dimension. Point estimates declined near 1.05–1.06 mmol/L, but the interaction did not retain statistical support after multiplicity adjustment and the crossing distribution was wide. This range is, therefore, a hypothesis for prospective evaluation, not a treatment threshold. The pattern is compatible with the nonlinear relationship between iCa and outcome reported in large ICU cohorts [7, 8, 34] and with the multifactorial, potentially adaptive nature of mild hypocalcemia during critical illness [4, 5, 35]. It also fits experimental evidence that excess extracellular calcium can amplify inflammatory and intracellular calcium signaling during sepsis [9, 36]. The higher observed risk of hypercalcemia suggests a potential safety trade-off, although post-index iCa was measured more frequently after early treatment. Intervals for severe arrhythmia, new RRT, and hospital mortality included the null. Together, these findings support randomized evaluation of a selective strategy based on iCa and vasopressor intensity, rather than automatic normalization across the entire 0.90–1.10 mmol/L range.

The exploratory clinical-context analyses did not identify multiplicity-adjusted variation by infection source, PCT, or pathogen group. The exploratory SOFA interaction followed the NEE pattern, with larger estimated differences at greater illness severity. This concordance suggests that the association between early calcium and hemodynamic recovery may vary with the severity of organ dysfunction. Because SOFA was not a prespecified effect modifier and overlaps with measured shock burden, the finding remains hypothesis-generating. Echocardiographic, hepatic, inflammatory, and microbiologic data characterize the cohort and support the expanded-covariate analysis, but their selective availability limits subgroup inference.

The study contributes an executable design for a definitive trial. Adults with sepsis, iCa 0.90–1.10 mmol/L, and no emergency indication could be randomized at result availability to calcium within 2 h or deferral through 6 h, with stratification by baseline NEE and iCa. Twenty-four-hour hemodynamic recovery and hypercalcemia would capture the early benefit–harm balance, accompanied by longer-term organ-support and mortality outcomes. Enrichment for vasopressor-treated patients and prespecified evaluation of continuous iCa heterogeneity would directly test the pattern observed here. Such a trial could determine whether a readily available drug accelerates resolution of septic circulatory failure in a defined, time-sensitive setting.

Several features define the scope of inference. Treatment was observational, and residual confounding by indication remains possible because clinicians may respond to hemodynamic information, electrocardiographic features, or bedside assessments incompletely captured in the electronic record; time-updated clone–censor weighting and strong measured-covariate balance reduce this concern without eliminating it. Data came from a single tertiary teaching hospital, and overlap weighting targets patients with clinical equipoise, so effects in institutions with different supplementation practices or near-deterministic treatment decisions require external assessment. The formal deferred strategy allowed treatment after 6 h, yet no deferred-pathway patient received calcium during 6–24 h; inference, therefore, concerns the observed early-versus-no-calcium pathway. The exact 24-h endpoint can be influenced by local vasopressor-weaning, sedation, and discharge practices. Calcium salt, dose, and repeat administration followed clinical care. Reliable structured data on parathyroid disease, vitamin D status, and bisphosphonate exposure were unavailable, while magnesium and phosphate were infrequently measured. Echocardiography, inflammatory markers, and microbiologic testing were selective; only 39 patients had recorded SARS-CoV-2 positivity, precluding a separate analysis. Safety events were uncommon, and post-index iCa was measured more often after early calcium, which may inflate the apparent hypercalcemia contrast. The SOFA, infection-source, PCT, and pathogen analyses were exploratory. Finally, the iCa interaction did not remain statistically supported after Holm correction, the crossing distribution was wide, and data near the iCa range boundaries were limited. The upper NEE range also contained fewer patients than the near-zero region. These constraints define the population and measurements required for confirmation.

## Conclusions

Among adults with sepsis and non-emergency iCa of 0.90–1.10 mmol/L, intravenous calcium initiated within 2 h was associated with a higher probability of being alive and vasopressor-free at 24 h than the observed deferred pathway, in which no patient received calcium through 24 h. Estimated differences were larger at higher baseline vasopressor doses. The iCa pattern remained exploratory and did not define a treatment threshold. Early treatment was also associated with a modestly higher observed risk of hypercalcemia. These findings support a randomized trial stratified by baseline iCa and vasopressor intensity.

## Supplementary Information

Supplementary Material 1

## Data Availability

The de-identified clinical data analyzed in this study are not publicly available because of patient-privacy and institutional data-governance restrictions. Qualified researchers may request access through the corresponding author. Access is subject to approval by Zigong Fourth People's Hospital and completion of an applicable data-use agreement. The analytic code is available from the corresponding author upon reasonable request.

## Abbreviations

ATO: Average treatment effect in the overlap population
CCW: Clone–censor–weight
CI: Confidence interval
CRP: C-reactive protein
ICU: Intensive care unit
iCa: Ionized calcium
LVEF: Left ventricular ejection fraction
MAP: Mean arterial pressure
MIMIC: Medical Information Mart for Intensive Care
NEE: Norepinephrine-equivalent dose
PCT: Procalcitonin
RECORD: REporting of studies Conducted using Observational Routinely collected health Data
RRT: Renal replacement therapy
SD: Standard deviation
SMD: Standardized mean difference
SOFA: Sequential Organ Failure Assessment
STROBE: Strengthening the Reporting of Observational Studies in Epidemiology
TARGET: Transparent Reporting of Observational Studies Emulating a Target Trial
TTE: Transthoracic echocardiography
