# Supplementary material for: Early versus deferred intravenous calcium in patients with sepsis and ionized hypocalcemia: a target-trial emulation
Source: J Intensive Care. 2026 Oct 13;14:110. doi: 10.1186/s40560-026-00953-9 (PMC13660333; doi:10.1186/s40560-026-00953-9)
Supplement: Supplementary file 1 [file 40560_2026_953_MOESM1_ESM.docx]

**Additional file 1**

***Early versus deferred intravenous calcium in patients with sepsis and ionized hypocalcemia: a target-trial emulation***

Yuhang Zhang¹, Guoying Liao², Chenghao Liu³, Guanyu Chen², Jianli Song², Yinsheng Liao², Lulu Zhang²*

**Affiliations:**

¹ Department of Anesthesiology, The Second Affiliated Hospital of Chongqing Medical University, Chongqing, 400010, China.

² Department of Anesthesiology, Zigong Fourth People's Hospital, Zigong, Sichuan, 643000, China.

³ Computer Center, Zigong Fourth People's Hospital, Zigong, Sichuan, 643000, China.

*Corresponding author: Lulu Zhang, Department of Anesthesiology, Zigong Fourth People's Hospital, Zigong, Sichuan, 643000, China. Email: zhangll.anes.zfph@suse.edu.cn

**Contents:**

Table S1. Target-trial specification and emulation

Table S2. Data sources, operational definitions, and eligibility algorithms

Table S3. Cohort, treatment-path, and clone flow

Table S4. Baseline missingness and multiple-imputation handling

Table S5. Complete covariate balance and weight diagnostics

Table S6. Additional baseline clinical characteristics and data availability

Table S7. Liver, inflammatory, and microbiologic characteristics

Table S8. Primary and sensitivity estimates

Table S9. Prespecified and exploratory effect-modification analyses

Table S10. Secondary outcomes and outcome ascertainment

Figure S1. Observed calcium-initiation timing and treatment support

Figure S2. Repeated-eligibility positivity audit

Figure S3. Baseline overlap and final CCW–ATO weight distributions

Figure S4. Complete covariate balance before and after weighting

Figure S5. Patient-level bootstrap stability

Figure S6. Time-discretization diagnostics

Figure S7. Heterogeneity support and uncertainty in the exploratory iCa crossing

Figure S8. Expanded-model balance and exploratory effect modification

**Table S1. Target-trial specification and emulation.**

| **Protocol component** | **Hypothetical target trial** | **Observational emulation** |
| --- | --- | --- |
| Eligibility | Adults in an ICU with sepsis-associated iCa 0.90–1.10 mmol/L and no emergency indication for calcium | First fully eligible event per patient in 2016–2025 high-resolution hospital data; n=5,993 |
| Strategies | Initiate intravenous calcium within 2 h versus withhold intravenous calcium through 6 h; care after 6 h unrestricted | Executed non-CRRT calcium gluconate or calcium chloride; orders alone did not define treatment; treatment after 6 h was neither required nor prohibited |
| Assignment | Random allocation at time zero | Each eligible patient was cloned into both strategies at time zero |
| Time zero | Availability of the qualifying iCa result | Final/corrected result datetime; specimen-collection anchoring was not used in the formal CCW estimand |
| Grace periods | 0–2 h for initiation; 0–6 h for deferral | Thirty-minute intervals; 60-minute discretization was a sensitivity analysis |
| Follow-up | From time zero through 24 h | Death before 24 h was retained as primary-outcome failure |
| Primary outcome | Alive and free of vasopressor infusion at 24 h | No positive-rate norepinephrine, epinephrine, vasopressin, or dopamine infusion at 24 h |
| Secondary outcomes | Short-term safety, organ support, and mortality | Hypercalcemia, severe arrhythmia, new RRT within 24 h, and hospital mortality |
| Causal contrast | Per-protocol early versus deferred strategy | Standardized strategy risks, RD, and RR |
| Target population | Eligible clinical-overlap population | Baseline ATO population rather than the whole-cohort average treatment effect |
| Artificial censoring | Censor a clone when its observed path becomes incompatible | Early clone censored if alive and untreated after 2 h; deferred clone censored at initiation before 6 h |
| Weights | Inverse adherence-path weighting | Discrete-time observed-path weights multiplied by baseline overlap tilting; untruncated primary weights |
| Missing data and inference | Complete measurement in the hypothetical trial | Primary inference used 2,000 patient bootstraps with refitting of both weight models; 20 chained-equation imputations with clustered analytic variance were complementary |

**Note.** The hypothetical protocol and its observational emulation are shown side by side. Treatment at 2–6 h was retained in the cloned population and subsequently censored from both strategies; it was not removed before cloning. ATO, average treatment effect in the overlap population; CCW, clone–censor–weight; CRRT, continuous renal replacement therapy; h, hours; iCa, ionized calcium; ICU, intensive care unit; n, number of patients; RD, risk difference; RR, risk ratio; RRT, renal replacement therapy.

**Table S2. Data sources, operational definitions, and eligibility algorithms.**

| **Domain** | **Variable or item** | **Operational definition** | **Timing or source** |
| --- | --- | --- | --- |
| Eligibility | Adult ICU stay | Age ≥18 years; qualifying result occurred during a documented ICU stay | Hospital and ICU records |
| Eligibility | Operational sepsis | At least two infection-evidence categories in the preceding 72 h plus SOFA ≥2 from past-only data | Antibiotic, microbiology, diagnosis/event, organ-function data |
| Eligibility | Index iCa | Final/corrected iCa result 0.90–1.10 mmol/L | Result datetime defined time zero |
| Eligibility | Prior calcium | Exclude executed intravenous calcium in the preceding 6 h | Medication administrations |
| Eligibility | Emergency indications | Exclude recent arrest/resuscitation or bypass, K≥6.0 mmol/L, massive transfusion, citrate-CRRT, active ECMO/MCS, or calcium-channel-blocker poisoning | Locked indication-specific look-back windows |
| Exposure | Early calcium strategy | Executed non-CRRT calcium gluconate or calcium chloride began from 0 through 2 h | Administration start time |
| Exposure | Deferred calcium strategy | No executed non-CRRT intravenous calcium from 0 through 6 h | Administration history |
| Exposure | Treatment at 2–6 h | Incompatible with both strategies after the applicable deviation time | Retained at cloning; artificially censored |
| Baseline | Age and sex | Age at admission; sex at birth | At admission |
| Baseline | Calendar, location, ICU type | Calendar year, deidentified within-institution administrative location code, and ICU category | At time zero |
| Baseline | SOFA | Total Sequential Organ Failure Assessment score from available past-only components; missing components scored 0 | Preceding 24 h |
| Baseline | iCa and ICU time | Index iCa and hours from ICU admission to time zero | At time zero |
| Baseline | Infection evidence | Number of distinct qualifying infection-evidence categories | Preceding 72 h |
| Baseline | pH | pH from the same specimen as index iCa and available by time zero | Same specimen; otherwise missing |
| Baseline | Lactate and potassium | Most recent eligible past-only lactate and maximum potassium | Locked laboratory windows |
| Baseline | Renal and hematologic laboratories | Creatinine maximum; platelet minimum; hemoglobin minimum; white-cell maximum | Locked past-only windows |
| Baseline | Hepatic/nutritional laboratories | Bilirubin maximum and albumin minimum | Locked past-only windows |
| Baseline | MAP, heart rate, GCS | Minimum MAP, maximum heart rate, and minimum GCS | Available before time zero |
| Baseline | NEE | Sum of active norepinephrine-equivalent vasopressor infusion rates | At time zero; µg/kg/min |
| Baseline | Organ support | Invasive ventilation and active RRT | At time zero |
| Baseline | Hydrocortisone/transfusion | Executed hydrocortisone and blood-product exposure | Pre-index locked windows |
| Time-updated | Treatment-path model | Current/recent iCa, pH, lactate, potassium, MAP, heart rate, NEE, ventilation, RRT, and transfusion when available | Known by interval start |
| Outcome | Hemodynamic recovery | Alive with no positive-rate norepinephrine, epinephrine, vasopressin, or dopamine at 24 h | Primary endpoint |
| Outcome | Hypercalcemia | Post-index iCa >1.30 mmol/L within 24 h | Requires a post-index iCa measurement |
| Outcome | Severe arrhythmia | New severe or treatment-requiring arrhythmia within 24 h | Rhythm/procedure/medication records |
| Outcome | New RRT | New renal replacement therapy initiated within 24 h | RRT/CRRT records |
| Outcome | Hospital mortality | Death before hospital discharge | Outcome and encounter records |

**Note.** All eligibility, exposure, covariate, and outcome definitions were applied by the study team after receipt of unscreened ICU-linked data. Only information available by the relevant decision or interval start entered treatment-path models. CRRT, continuous renal replacement therapy; ECMO, extracorporeal membrane oxygenation; GCS, Glasgow Coma Scale; h, hours; iCa, ionized calcium; ICU, intensive care unit; K, potassium; MAP, mean arterial pressure; MCS, mechanical circulatory support; NEE, norepinephrine-equivalent dose; RRT, renal replacement therapy; SOFA, Sequential Organ Failure Assessment.

**Table S3. Cohort, treatment-path, and clone flow.**

| **Stage** | **Rule or observed history** | **n** |
| --- | --- | --- |
| Eligibility support | Six-hour-spaced eligible decisions | 17,644 |
| Formal population | Unique patients contributing their first eligible decision | 5,993 |
| Observed path | Calcium initiated within 0–2 h | 1,538 |
| Observed path | Calcium initiated after 2 h but before 6 h | 147 |
| Observed path | No calcium initiation through 6 h | 4,308 |
| Observed path | Calcium initiated after 6 h but within 24 h among the deferred pathway | 0 |
| Observed path | No calcium initiation through 24 h among the deferred pathway | 4,308 |
| Cloning | Initial clones (two per eligible patient) | 11,986 |
| Endpoint-compatible | Early-strategy clones | 1,539 |
| Endpoint-compatible | Deferred-strategy clones | 4,308 |
| Artificial censoring | Early-strategy clones censored after deviation | 4,454 |
| Artificial censoring | Deferred-strategy clones censored after deviation | 1,685 |
| Grace-period deaths | Deaths retained before the early deadline | 1 |
| Grace-period deaths | Deaths retained before the deferred deadline | 7 |
| Hazard model | At-risk interval rows / calcium initiations | 55,381 / 1,685 |

**Note.** Counts describe the locked first-eligible CCW analysis. The early endpoint-compatible count exceeds observed 0–2-h initiations by one because a death during the early grace period remained compatible and was retained as an outcome failure. Histories treated at 2–6 h were cloned and later artificially censored under both strategies. The deferred strategy required no calcium through 6 h; in the observed data, none of the 4,308 deferred-pathway patients initiated intravenous calcium during 6–24 h. Thus, the observed comparator was no calcium through 24 h rather than mandatory later treatment. CCW, clone–censor–weight; h, hours; n, number of decisions, patients, clones, or interval rows as specified.

**Table S4. Baseline missingness and multiple-imputation handling.**

| **Baseline variable** | **Missing n** | **Missing, %** | **Handling** |
| --- | --- | --- | --- |
| Age | 0 | 0.0% | Not imputed |
| Female sex | 0 | 0.0% | Not imputed |
| Calendar year | 0 | 0.0% | Not imputed |
| Administrative location | 0 | 0.0% | Not imputed |
| ICU type | 0 | 0.0% | Not imputed |
| SOFA score | 0 | 0.0% | Not imputed |
| Index iCa | 0 | 0.0% | Not imputed |
| Hours since ICU admission | 0 | 0.0% | Not imputed |
| Infection-evidence categories | 0 | 0.0% | Not imputed |
| Same-specimen pH | 1,603 | 26.7% | PMM in 20 MICE sets; median + missing indicator in bootstrap |
| Lactate | 1,411 | 23.5% | PMM in 20 MICE sets; median + missing indicator in bootstrap |
| Potassium | 1,307 | 21.8% | PMM in 20 MICE sets; median + missing indicator in bootstrap |
| Creatinine | 1,128 | 18.8% | PMM in 20 MICE sets; median + missing indicator in bootstrap |
| Platelet count | 1,176 | 19.6% | PMM in 20 MICE sets; median + missing indicator in bootstrap |
| Bilirubin | 2,396 | 40.0% | PMM in 20 MICE sets; median + missing indicator in bootstrap |
| Albumin | 2,414 | 40.3% | PMM in 20 MICE sets; median + missing indicator in bootstrap |
| Hemoglobin | 1,208 | 20.2% | PMM in 20 MICE sets; median + missing indicator in bootstrap |
| White blood cell count | 1,250 | 20.9% | PMM in 20 MICE sets; median + missing indicator in bootstrap |
| Mean arterial pressure | 11 | 0.2% | PMM in 20 MICE sets; median + missing indicator in bootstrap |
| Heart rate | 58 | 1.0% | PMM in 20 MICE sets; median + missing indicator in bootstrap |
| Glasgow Coma Scale | 1,764 | 29.4% | PMM in 20 MICE sets; median + missing indicator in bootstrap |
| Norepinephrine-equivalent dose | 0 | 0.0% | Not imputed |
| Invasive ventilation | 0 | 0.0% | Not imputed |
| Active RRT | 0 | 0.0% | Not imputed |
| Hydrocortisone | 0 | 0.0% | Not imputed |
| Blood transfusion | 0 | 0.0% | Not imputed |

**Note.** Missingness was evaluated among all 5,993 first-eligible patients before imputation. Exposure, time zero, death, and outcomes were never imputed. Continuous incomplete baseline covariates were multiply imputed in 20 chained-equation data sets using predictive mean matching; observed time-updated measurements superseded imputed baseline carry-forward values. The 2,000-replicate bootstrap used deterministic median/mode replacement with missing indicators and refitted both weight models in every replicate. All 20 imputation data sets completed. ICU, intensive care unit; iCa, ionized calcium; MICE, multiple imputation by chained equations; PMM, predictive mean matching; RRT, renal replacement therapy; SOFA, Sequential Organ Failure Assessment.

**Table S5. Complete covariate balance and weight diagnostics.**

| **Covariate or model term** | **SMD before** | **SMD after** | **\|SMD\| after** |
| --- | --- | --- | --- |
| Age | +0.047 | +0.013 | 0.013 |
| Female sex | -0.057 | -0.046 | 0.046 |
| SOFA score | +0.523 | +0.013 | 0.013 |
| Index iCa | -0.072 | -0.004 | 0.004 |
| Hours since ICU admission | -0.847 | +0.041 | 0.041 |
| Infection-evidence categories | -0.161 | +0.003 | 0.003 |
| Same-specimen pH | -0.163 | -0.017 | 0.017 |
| Lactate | +0.055 | -0.014 | 0.014 |
| Potassium | +0.081 | +0.024 | 0.024 |
| Creatinine | -0.155 | -0.014 | 0.014 |
| Platelet count | +0.109 | +0.025 | 0.025 |
| Bilirubin | +0.083 | -0.028 | 0.028 |
| Albumin | -0.254 | +0.033 | 0.033 |
| Hemoglobin | -0.065 | -0.006 | 0.006 |
| White blood cell count | +0.186 | +0.009 | 0.009 |
| Mean arterial pressure | +0.088 | -0.009 | 0.009 |
| Heart rate | -0.131 | -0.043 | 0.043 |
| Glasgow Coma Scale | +0.201 | +0.050 | 0.050 |
| Norepinephrine-equivalent dose | +0.372 | +0.027 | 0.027 |
| Invasive ventilation | +0.208 | +0.042 | 0.042 |
| Active RRT | -0.102 | +0.011 | 0.011 |
| Hydrocortisone | +0.011 | -0.035 | 0.035 |
| Blood transfusion | -0.060 | -0.040 | 0.040 |
| Administrative location A | +0.058 | +0.011 | 0.011 |
| Administrative location B | -0.058 | -0.011 | 0.011 |
| Calendar year 2017 | -0.126 | +0.043 | 0.043 |
| Calendar year 2018 | -0.041 | +0.007 | 0.007 |
| Calendar year 2019 | +0.012 | -0.055 | 0.055 |
| Calendar year 2020 | +0.040 | +0.003 | 0.003 |
| Calendar year 2021 | +0.042 | -0.049 | 0.049 |
| Calendar year 2022 | +0.058 | -0.038 | 0.038 |
| Calendar year 2023 | +0.063 | -0.017 | 0.017 |
| Calendar year 2024 | +0.091 | +0.087 | 0.087 |
| Calendar year 2025 | +0.080 | +0.039 | 0.039 |
| Emergency ICU | +0.033 | +0.022 | 0.022 |
| Medical ICU | +0.054 | -0.004 | 0.004 |
| Mixed ICU | +0.029 | -0.015 | 0.015 |
| Neurological ICU | -0.204 | -0.001 | 0.001 |
| Surgical ICU | -0.009 | +0.013 | 0.013 |
| Trauma ICU | -0.187 | -0.001 | 0.001 |

**Note.** Signed SMDs compare observed early-compatible with deferred-compatible histories; weighted values use final CCW–ATO weights. The largest weighted |SMD| was 0.087, below the prespecified 0.10 threshold. Early weights: median 1.292, 99th percentile 14.64, maximum 27.27, ESS 632. Deferred weights: median 0.099, 99th percentile 0.781, maximum 1.989, ESS 1936. The denominator initiation hazard had 55,381 rows and 1,685 events; its 99th percentile was 0.352. Administrative locations A and B are deidentified location codes within Zigong Fourth People's Hospital. ATO, average treatment effect in the overlap population; CCW, clone–censor–weight; ESS, effective sample size; iCa, ionized calcium; ICU, intensive care unit; RRT, renal replacement therapy; SMD, standardized mean difference; SOFA, Sequential Organ Failure Assessment.

**Table S6. Additional baseline clinical characteristics and data availability.**

| **Domain** | **Characteristic** | **Early pathway** | **Deferred pathway** | **Availability** |
| --- | --- | --- | --- | --- |
| Infection source | Pulmonary source | 415 (27.0%) | 1,318 (30.6%) | Complete |
| Infection source | Urinary source | 243 (15.8%) | 681 (15.8%) | Complete |
| Infection source | Intra-abdominal source | 376 (24.4%) | 1,241 (28.8%) | Complete |
| Infection source | Skin or soft-tissue source | 231 (15.0%) | 668 (15.5%) | Complete |
| Infection source | Multiple recorded sources | 261 (17.0%) | 887 (20.6%) | Complete |
| Infection source | Other or undetermined source | 568 (36.9%) | 1,493 (34.7%) | Complete |
| Recorded comorbidity | Active malignancy | 157 (10.2%) | 727 (16.9%) | Complete |
| Recorded comorbidity | Heart failure | 179 (11.6%) | 719 (16.7%) | Complete |
| Recorded comorbidity | Chronic kidney disease | 151 (9.8%) | 648 (15.0%) | Complete |
| Recorded comorbidity | Cirrhosis | 151 (9.8%) | 706 (16.4%) | Complete |
| Recorded comorbidity | Diabetes mellitus | 157 (10.2%) | 669 (15.5%) | Complete |
| Mineral metabolism | Magnesium, mg/dL | 1.89 (1.86–1.92); n=279 | 1.90 (1.86–1.93); n=999 | Pre-index value |
| Mineral metabolism | Phosphate, mg/dL | 3.21 (3.08–3.30); n=287 | 3.21 (3.10–3.31); n=953 | Pre-index value |
| Cardiac assessment | Baseline TTE available | 1,070 (69.6%) | 2,726 (63.3%) | Observed availability |
| Cardiac assessment | LVEF, % | 54.6 (48.1–61.0); n=1,070 | 56.0 (49.8–61.6); n=2,726 | Among measured |
| Cardiac assessment | LVEF category <40% | 112 (7.3%) | 247 (5.7%) | Percentage of pathway |
| Cardiac assessment | LVEF category 40–49% | 223 (14.5%) | 446 (10.4%) | Percentage of pathway |
| Cardiac assessment | LVEF category ≥50% | 735 (47.8%) | 2,033 (47.2%) | Percentage of pathway |
| Cardiac assessment | LVEF category Missing | 468 (30.4%) | 1,582 (36.7%) | Percentage of pathway |

**Note.** Values are n (%) or median (interquartile range), as indicated, among the observed early and deferred pathways. Infection-source categories were not mutually exclusive. TTE was selected as the nearest qualifying examination within the prespecified pre-index window; absence of TTE was retained as missing rather than interpreted as normal cardiac function. Reliable structured variables for parathyroid disease, vitamin D status, and bisphosphonate exposure were unavailable. CKD, chronic kidney disease; h, hours; LVEF, left ventricular ejection fraction; TTE, transthoracic echocardiography.

**Table S7. Liver, inflammatory, and microbiologic characteristics.**

| **Domain** | **Characteristic** | **Early pathway** | **Deferred pathway** | **Availability** |
| --- | --- | --- | --- | --- |
| Laboratory | Alanine aminotransferase, U/L | 39.17 (25.44–59.71); n=1,299 | 31.06 (21.01–47.56); n=3,506 | 84.5% / 81.4% |
| Laboratory | Aspartate aminotransferase, U/L | 42.17 (26.46–68.52); n=1,314 | 34.04 (22.44–53.22); n=3,503 | 85.4% / 81.3% |
| Laboratory | Total bilirubin, mg/dL | 1.11 (0.74–1.70); n=1,440 | 0.91 (0.63–1.37); n=3,968 | 93.6% / 92.1% |
| Laboratory | Albumin, g/L | 30.55 (26.97–34.26); n=1,424 | 32.08 (28.65–35.40); n=3,912 | 92.6% / 90.8% |
| Laboratory | International normalized ratio | 1.18 (1.08–1.30); n=1,382 | 1.12 (1.03–1.23); n=3,803 | 89.9% / 88.3% |
| Laboratory | White blood cell count, 10⁹/L | 11.29 (9.11–14.42); n=1,480 | 10.40 (8.25–13.24); n=4,085 | 96.2% / 94.8% |
| Laboratory | C-reactive protein, mg/L | 106.55 (68.62–163.13); n=1,304 | 83.93 (57.74–125.38); n=3,484 | 84.8% / 80.9% |
| Laboratory | Procalcitonin, ng/mL | 1.27 (0.63–2.64); n=1,294 | 0.82 (0.44–1.57); n=3,523 | 84.1% / 81.8% |
| Laboratory | Interleukin-6, pg/mL | 36.81 (15.95–79.59); n=526 | 22.85 (11.55–49.56); n=1,444 | 34.2% / 33.5% |
| Pathogen category | Not tested | 409 (26.6%) | 1,168 (27.1%) | All patients classified |
| Pathogen category | Tested; no relevant pathogen | 512 (33.3%) | 1,465 (34.0%) | All patients classified |
| Pathogen category | Bacterial only | 516 (33.6%) | 1,341 (31.1%) | All patients classified |
| Pathogen category | Fungal only | 45 (2.9%) | 133 (3.1%) | All patients classified |
| Pathogen category | Viral only | 25 (1.6%) | 91 (2.1%) | All patients classified |
| Pathogen category | Multiple pathogen domains | 31 (2.0%) | 110 (2.6%) | All patients classified |
| Detected viral target | SARS-CoV-2 | 6 | 33 | Positive patients |
| Detected viral target | Influenza A | 10 | 25 | Positive patients |
| Detected viral target | Influenza B | 9 | 38 | Positive patients |
| Detected viral target | Respiratory syncytial virus | 5 | 31 | Positive patients |
| Detected viral target | Human metapneumovirus | 12 | 39 | Positive patients |

**Note. Laboratory values are the most recent finalized or corrected results available within 24 h before time zero, summarized without imputation as median (interquartile range); availability percentages are shown as early/deferred. Pathogen categories used microbiology and viral testing recorded before time zero; the six categories are mutually exclusive. Organism and viral-target rows are descriptive because testing was selective and low-frequency strata did not support reliable organism-specific causal comparisons. ALT, alanine aminotransferase; AST, aspartate aminotransferase; CRP, C-reactive protein; IL-6, interleukin-6; INR, international normalized ratio; PCT, procalcitonin; SARS-CoV-2, severe acute respiratory syndrome coronavirus 2; WBC, white blood cell count.**

**Table S8. Primary and sensitivity estimates.**

| **Analysis** | **Early risk** | **Deferred risk** | **RD, pp (95% CI)** | **RR (95% CI)** | **Successful runs** |
| --- | --- | --- | --- | --- | --- |
| Deterministic 30-min estimate | 69.8% | 61.0% | +8.77 | 1.144 | Not applicable |
| Twenty-imputation analytic inference | 69.8% | 60.7% | +9.11 (+4.94 to +13.27) | 1.150 (1.080 to 1.224) | 20 imputations |
| Primary 30-min bootstrap | 69.9% | 61.1% | +8.69 (+4.66 to +12.84) | 1.142 (1.074 to 1.214) | 2,000/2,000 |
| Expanded-covariate 30-min bootstrap | 70.1% | 61.2% | +8.89 (+5.04 to +13.03) | 1.145 (1.082 to 1.217) | 2,000/2,000 |
| Weights truncated at 0.5th/99.5th percentiles | — | — | +8.41 (+4.57 to +12.40) | 1.138 (1.073 to 1.208) | 2,000/2,000 |
| Weights truncated at 1st/99th percentiles | — | — | +8.21 (+4.40 to +12.14) | 1.134 (1.070 to 1.202) | 2,000/2,000 |
| Weights truncated at 2.5th/97.5th percentiles | — | — | +7.83 (+4.24 to +11.57) | 1.127 (1.067 to 1.192) | 2,000/2,000 |
| Exclude ICU discharge before relevant grace endpoint | — | — | +9.02 (+4.96 to +13.12) | 1.148 (1.080 to 1.221) | 2,000/2,000 |
| Sixty-minute discretization | 74.0% | 60.7% | +13.20 (+8.80 to +17.42) | 1.218 (1.142 to 1.293) | 2,000/2,000 |

**Note.** The outcome was being alive and free of positive-rate vasopressor infusion at 24 h. RD values are percentage points; parentheses contain two-sided 95% intervals. The primary robustness intervals are percentile intervals from patient-level bootstraps in which both treatment-path and baseline-overlap models were refitted. The reviewer-requested expanded model additionally included magnesium, phosphate, LVEF, PCT, CRP, AST, INR, active malignancy, and infection-source terms with missingness indicators where applicable; its maximum weighted |SMD| was 0.090. Analytic multiple-imputation intervals used patient-clustered variance pooled with Rubin's rules. AST, aspartate aminotransferase; ATO, average treatment effect in the overlap population; CCW, clone–censor–weight; CI, confidence interval; CRP, C-reactive protein; ICU, intensive care unit; INR, international normalized ratio; LVEF, left ventricular ejection fraction; min, minutes; PCT, procalcitonin; RD, risk difference; RR, risk ratio; SMD, standardized mean difference.

**Table S9. Prespecified and exploratory effect-modification analyses.**

| **Analysis** | **Modifier or value** | **df** | **Estimate** | **95% interval** | **P** | **Holm P** |
| --- | --- | --- | --- | --- | --- | --- |
| Prespecified interaction | Index iCa | 3 | χ²=8.78 | — | 0.032 | 0.097 |
| Prespecified interaction | Same-specimen pH | 1 | χ²=0.43 | — | 0.511 | 1.000 |
| Prespecified interaction | Lactate | 1 | χ²=0.10 | — | 0.757 | 1.000 |
| Prespecified interaction | Baseline NEE | 1 | χ²=25.27 | — | 5.00 × 10⁻⁷ | 2.00 × 10⁻⁶ |
| Exploratory interaction | SOFA score | 3 | χ²=18.41 | — | 3.62 × 10⁻⁴ | — |
| Exploratory interaction | Source: pulmonary | 1 | χ²=0.04 | — | 0.841 | 1.000 |
| Exploratory interaction | Source: urinary | 1 | χ²=3.69 | — | 0.055 | 0.219 |
| Exploratory interaction | Source: intra-abdominal | 1 | χ²=0.09 | — | 0.760 | 1.000 |
| Exploratory interaction | Source: skin or soft tissue | 1 | χ²=0.02 | — | 0.877 | 1.000 |
| Exploratory interaction | Baseline PCT (continuous spline) | 3 | χ²=4.72 | — | 0.193 | 0.386 |
| Exploratory interaction | Pre-index pathogen group | 2 | χ²=2.60 | — | 0.273 | 0.386 |
| Prespecified marginal curve | NEE=0.00 µg/kg/min | — | RD=-0.42 pp | -5.48 to +4.24 | — | — |
| Prespecified marginal curve | NEE=0.10 µg/kg/min | — | RD=+8.64 pp | +4.29 to +12.81 | — | — |
| Prespecified marginal curve | NEE=0.20 µg/kg/min | — | RD=+19.58 pp | +13.60 to +25.26 | — | — |
| Prespecified marginal curve | NEE=0.30 µg/kg/min | — | RD=+29.24 pp | +20.69 to +37.57 | — | — |
| Prespecified marginal curve | iCa=0.95 mmol/L | — | RD=+12.26 pp | +4.94 to +19.12 | — | — |
| Prespecified marginal curve | iCa=1.00 mmol/L | — | RD=+12.39 pp | +6.96 to +17.81 | — | — |
| Prespecified marginal curve | iCa=1.05 mmol/L | — | RD=+3.81 pp | -4.00 to +10.48 | — | — |
| Prespecified marginal curve | iCa=1.10 mmol/L | — | RD=-13.48 pp | -32.50 to +4.78 | — | — |
| Exploratory crossing | Conditional on an in-range crossing | — | 1.058 mmol/L | 0.903 to 1.086 | — | — |
| Exploratory marginal curve | SOFA=2 | — | RD=-1.63 pp | -10.24 to +5.38 | — | — |
| Exploratory marginal curve | SOFA=6 | — | RD=+2.61 pp | -4.78 to +8.90 | — | — |
| Exploratory marginal curve | SOFA=8 | — | RD=+14.30 pp | +8.20 to +20.59 | — | — |
| Exploratory marginal curve | SOFA=10 | — | RD=+21.87 pp | +14.72 to +29.12 | — | — |
| Exploratory marginal curve | SOFA=12 | — | RD=+25.31 pp | +15.07 to +35.30 | — | — |
| Exploratory marginal curve | SOFA=14 | — | RD=+25.63 pp | +5.24 to +43.11 | — | — |
| Exploratory marginal curve | PCT=0.25 ng/mL | — | RD=+2.47 pp | -6.12 to +10.28 | — | — |
| Exploratory marginal curve | PCT=1 ng/mL | — | RD=+7.81 pp | +0.91 to +14.06 | — | — |
| Exploratory marginal curve | PCT=2 ng/mL | — | RD=+15.46 pp | +8.35 to +22.51 | — | — |
| Exploratory marginal curve | PCT=5 ng/mL | — | RD=+16.82 pp | +8.10 to +25.24 | — | — |
| Exploratory marginal curve | PCT=10 ng/mL | — | RD=+11.15 pp | -3.54 to +26.11 | — | — |
| Exploratory contrast | Pulmonary: present minus absent | — | ΔRD=-1.29 pp | -12.03 to +8.25 | 0.772 | 1.000 |
| Exploratory contrast | Urinary: present minus absent | — | ΔRD=-11.10 pp | -23.79 to +1.80 | 0.082 | 0.328 |
| Exploratory contrast | Intra-abdominal: present minus absent | — | ΔRD=-2.06 pp | -12.69 to +7.12 | 0.683 | 1.000 |
| Exploratory contrast | Skin or soft tissue: present minus absent | — | ΔRD=-1.33 pp | -14.01 to +10.85 | 0.833 | 1.000 |
| Exploratory stratum estimate | No relevant pathogen | — | RD=+12.70 pp | +6.26 to +18.71 | — | — |
| Exploratory stratum estimate | Bacterial only | — | RD=+12.42 pp | +6.18 to +18.88 | — | — |
| Exploratory stratum estimate | Other or multiple domains | — | RD=-2.39 pp | -22.51 to +16.62 | — | — |

**Note.** The four prespecified interaction tests formed one family and were adjusted by the Holm method. Reviewer-requested analyses of SOFA score, infection source, PCT, and pathogen group were exploratory; the four infection-source tests formed one Holm family and the PCT/pathogen tests formed another. Marginal estimates are CCW–ATO risk differences from 2,000 patient bootstraps with refitted weights. An in-range iCa crossing occurred in 1,903/2,000 replicates; the reported crossing interval is conditional on a crossing and does not establish a clinical threshold. ATO, average treatment effect in the overlap population; CCW, clone–censor–weight; CI, confidence interval; df, degrees of freedom; iCa, ionized calcium; NEE, norepinephrine-equivalent dose; PCT, procalcitonin; pp, percentage points; RD, risk difference; SOFA, Sequential Organ Failure Assessment.

**Table S10. Secondary outcomes and outcome ascertainment.**

| **Outcome** | **Events: early** | **Events: deferred** | **Early risk** | **Deferred risk** | **RD, pp (95% CI)** | **RR (95% CI)** | **Ascertainment** |
| --- | --- | --- | --- | --- | --- | --- | --- |
| Hypercalcemia within 24 h | 22 | 5 | 1.38% | 0.08% | +1.29 (+0.64 to +2.05) | 17.460 (5.701 to 472.113) | Post-index iCa observed: 85.8% vs 78.3% |
| Severe arrhythmia within 24 h | 16 | 57 | 0.92% | 1.44% | -0.52 (-1.25 to +0.23) | 0.635 (0.281 to 1.207) | Rhythm ascertainment from structured records |
| New RRT within 24 h | 38 | 64 | 2.63% | 2.12% | +0.50 (-0.88 to +2.07) | 1.232 (0.652 to 2.153) | RRT initiation from structured records |
| Hospital mortality | 212 | 417 | 12.20% | 14.20% | -1.97 (-4.62 to +1.07) | 0.860 (0.694 to 1.080) | Hospital outcome available |

**Note.** Event counts are unweighted among endpoint-compatible clones; strategy risks and contrasts use the primary CCW–ATO weights. Parentheses contain 95% patient-bootstrap intervals from 2,000 refitted-weight replicates. The hypercalcemia RR is unstable because the deferred event risk was very low, and its ascertainment is vulnerable to differential post-index iCa measurement. Secondary outcomes were exploratory. ATO, average treatment effect in the overlap population; CCW, clone–censor–weight; CI, confidence interval; iCa, ionized calcium; RD, risk difference; RR, risk ratio; RRT, renal replacement therapy.

**Figure S1. Observed calcium-initiation timing and treatment support.** Among 5,993 first-eligible patients, Panel A shows executed intravenous-calcium initiation times through 6 h after the final/corrected index iCa result; the dashed line marks 2 h and blue shading marks the 2–6-h interval. Panel B shows the observed 30-min initiation hazard among patients alive and untreated at each interval start. Panel C shows the corresponding number alive and untreated. Early calcium was defined as initiation within 2 h; deferred calcium as no initiation through 6 h. h, hours; iCa, ionized calcium; min, minutes.

**Figure S2. Repeated-eligibility positivity audit.** Panels A and B show the early-initiation proportion and eligible-decision count across the first eight six-hour-spaced decisions per patient. Panels C and D compare the repeated-entry diagnostic with the locked first-eligible analysis. The dashed line in Panel C marks the prespecified |SMD|=0.10 balance criterion. The repeated-entry early-strategy ESS is approximate because that diagnostic was stopped for non-identifiability; it is not an effect estimate. CCW, clone–censor–weight; ESS, effective sample size; SMD, standardized mean difference.

**Figure S3. Baseline overlap and final CCW–ATO weight distributions.** Panel A shows the baseline propensity for the early strategy among endpoint-compatible early and deferred clones. Panel B shows the distribution of final CCW–ATO weights on a log10 scale; horizontal bars mark medians. Panel C reports strategy-specific effective sample sizes. Early calcium was initiation within 2 h; deferred calcium was no initiation through 6 h. ATO, average treatment effect in the overlap population; CCW, clone–censor–weight; ESS, effective sample size.

**Figure S4. Complete covariate balance before and after weighting.** Absolute standardized mean differences are shown for all 40 terms in the baseline overlap model before and after final CCW–ATO weighting. Lines connect the unweighted and weighted values for the same term; the dashed vertical line marks the prespecified 0.10 criterion. The maximum weighted absolute standardized mean difference was 0.087. Administrative locations A and B are deidentified location codes within Zigong Fourth People's Hospital. ATO, average treatment effect in the overlap population; CCW, clone–censor–weight; iCa, ionized calcium; ICU, intensive care unit; SMD, standardized mean difference; SOFA, Sequential Organ Failure Assessment.

**Figure S5. Patient-level bootstrap stability.** Panels A and B show the empirical distributions of the risk difference and risk ratio across 2,000 patient-level bootstrap replicates. Solid colored lines mark bootstrap medians, shaded bands mark 95% percentile intervals, and gray dashed lines mark the null. Panel C relates the risk difference to early-strategy effective sample size; the two negative replicates are retained and highlighted. Both treatment-path and overlap models were refitted in every replicate. CI, confidence interval; ESS, effective sample size; RD, risk difference; RR, risk ratio.

**Figure S6. Time-discretization diagnostics.** Panel A compares final weight distributions under 30-min and 60-min discretization; values are shown on a log10 scale and outliers are suppressed only from the box-plot rendering. Panel B compares strategy-specific effective sample sizes; color denotes strategy and hatching denotes 60-min intervals. Panel C shows risk differences with 95% patient-bootstrap intervals and the maximum weighted absolute standardized mean difference for each specification. ATO, average treatment effect in the overlap population; CCW, clone–censor–weight; ESS, effective sample size; min, minutes; pp, percentage points; SMD, standardized mean difference.

**Figure S7. Heterogeneity support and uncertainty in the exploratory iCa crossing.** Panels A and B show endpoint-compatible clone counts as bars and effective sample sizes as diamonds across prespecified baseline norepinephrine-equivalent-dose and index-ionized-calcium strata. Panel C shows the conditional distribution of in-range iCa zero crossings across 2,000 refitted-weight bootstraps; crossings occurred in 1,903 replicates, with conditional median 1.058 mmol/L. This distribution quantifies uncertainty and does not validate a clinical threshold. ESS, effective sample size; iCa, ionized calcium; NEE, norepinephrine-equivalent dose.

**Figure S8. Expanded-model balance and exploratory effect modification.** Panel A shows balance for the additional covariates in the reviewer-requested expanded CCW model; connected points are the unweighted and weighted absolute standardized mean differences and the dashed line marks 0.10. Panels B and C show exploratory marginal risk-difference curves across baseline SOFA score and PCT, with 95% patient-bootstrap intervals. Panel D shows differences in risk difference for the presence versus absence of four infection sources. Panel E shows stratum-specific risk differences by pre-index pathogen group. The SOFA, infection-source, PCT, and pathogen analyses were exploratory. CCW, clone–censor–weight; CRP, C-reactive protein; INR, international normalized ratio; LVEF, left ventricular ejection fraction; PCT, procalcitonin; pp, percentage points; RD, risk difference; SMD, standardized mean difference; SOFA, Sequential Organ Failure Assessment.
